# Symbiont interactions with non-native hosts limit the formation of new symbioses

**DOI:** 10.1186/s12862-018-1143-z

**Published:** 2018-03-12

**Authors:** Natalie Niepoth, Jacintha Ellers, Lee M. Henry

**Affiliations:** 1 Animal Ecology, Department of Ecological Science, VU University Amsterdam, De Boelelaan 1085, 1081 HV Amsterdam, The Netherlands; 2 Present address: Department of Ecology, Evolution and Environmental Biology, Columbia University, New York, NY USA; 3 Present address: School of Biological and Chemical Sciences, Queen Mary University of London, Mile End Road, E1 4NS, London, England

**Keywords:** Bacterial mutualism, Facultative symbiosis, Horizontal transfer, Co-evolution

## Abstract

**Background:**

Facultative symbionts are common in eukaryotes and can provide their hosts with significant fitness benefits. Despite the advantage of carrying these microbes, they are typically only found in a fraction of the individuals within a population and are often non-randomly distributed among host populations. It is currently unclear why facultative symbionts are only found in certain host individuals and populations. Here we provide evidence for a mechanism to help explain this phenomenon: that when symbionts interact with non-native host genotypes it can limit the horizontal transfer of symbionts to particular host lineages and populations of related hosts.

**Results:**

Using reciprocal transfections of the facultative symbiont *Hamiltonella defensa* into different pea aphid clones, we demonstrate that particular symbiont strains can cause high host mortality and inhibit offspring production when injected into aphid clones other than their native host lineage. However, once established, the symbiont’s ability to protect against parasitoids was not influenced by its origin. We then demonstrate that *H. defensa* is also more likely to establish a symbiotic relationship with aphid clones from a plant-adapted population (biotype) that typically carry *H. defensa* in nature, compared to clones from a biotype that does not normally carry this symbiont.

**Conclusions:**

These results provide evidence that certain aphid lineages and populations of related hosts are predisposed to establishing a symbiotic relationship with *H. defensa*. Our results demonstrate that host-symbiont genotype interactions represent a potential barrier to horizontal transmission that can limit the spread of symbionts, and adaptive traits they carry, to certain host lineages.

**Electronic supplementary material:**

The online version of this article (10.1186/s12862-018-1143-z) contains supplementary material, which is available to authorized users.

## Background

Over the history of eukaryotes, symbiotic unions with microbes have resulted in key innovations that have profoundly influenced their host’s evolution. Many eukaryotes depend on obligate bacterial symbionts to synthesize nutrients absent in their diets and to perform other essential functions [1]. In the last few decades, a large number of facultative associations have been discovered where a symbiont is not essential for host survival but can increase host fitness in response to certain environmental stresses [2].

Host specificity is common in endosymbiotic partnerships and is found in many host-symbiont associations, including coral – *Symbiodinium*, grass – endophyte, legume – rhizobia, and human – gut microbiota [3–6]. Within insects, facultative symbionts are often only found in a fraction of the individuals within a population and can be non-randomly distributed across populations (e.g. [7–9]). It is currently unclear what explains the distribution of facultative symbionts in nature, but it has been suggested that symbiont-conferred benefits to hosts may explain their high frequency in certain ecological niches [10, 11].

Some of the best examples of facultative symbionts providing hosts with advantageous traits are found in the endosymbiotic bacteria of insects. These microbes can confer an array of benefits that have the potential to increase their host’s fitness in certain ecological conditions [12]. For example, facultative symbionts can benefit insects by protecting them from natural enemies, buffering against heat stress, aiding in plant-feeding, providing nutrients, and even detoxifying pesticides (reviewed in [13]). However, symbionts can also impose a fitness cost on the host [14, 15]. Although symbiont-conferred costs and benefits have been well documented in stable infections, the consequences of symbiont infections during the initial establishment period that directly follows horizontal transfer to a new host are less well-characterized. Studies that have investigated early establishment of symbioses in insects have shown that horizontal transfer can be influenced by relatedness between host species [16, 17], can vary in success when transferred between plant-adapted populations within the same host species [18], and can cause negative fitness consequences to the new host when horizontally transferred across species boundaries [19–22].

The pea aphid (*Acyrthosiphon pisum*) is a valuable model for studying the spread of facultative symbionts. Populations of the pea aphid are adapted to at least eleven different food-plants and form a complex of genetically differentiated host races or “biotypes” [23]. Surveys of facultative symbionts have clearly shown that certain species of bacteria are strongly associated with different aphid biotypes. For example, the symbiont *Hamiltonella defensa*, which is known for its ability to protect aphids from attack by parasitoid wasps [24], is found at high frequencies in aphid populations that feed on the plants *Medicago sativa*, *Ononis spinosa,* and *Lotus pedunculatus* [25], but are rarely found infecting pea aphids that feed on *Lotus corniculatus* [9]. A recent study has also shown that aphids belonging to biotypes that typically harbor *H. defensa* tend to carry genetically distinct symbiont strains. In particular, there are specific clades of *H. defensa* strains associated with aphids feeding on *M. sativa*, *O. spinosa* and *L. pedunculatus* [9].

One possible mechanism explaining *H. defensa*’s presence in certain aphid lineages over others is that particular combinations of symbiont strains and host genotypes may provide greater fitness benefits, or are less costly to the host [9], which could indicate a preadaptation of certain host genotypes to accommodate certain symbiont genotypes. This mechanism may also play a role in explaining the presence of symbionts in populations of genetically related host insects or biotypes if, for example, a symbiont provides greater benefits or is more likely to establish a symbiosis with individuals from one biotype over another.

Here we test if host and symbiont genotypes interact in a way that prevents symbioses from forming, by either i) limiting the establishment of *H. defensa* strains to particular host lineages, or ii) providing greater fitness benefits or costs to certain host lineages over others, which may impact their persistence in nature. First, we test if *H. defensa* strains provide greater fitness benefits or are less costly when introduced into certain aphid clones over others, and if native host lineages receive greater benefit than non-native host lineages. In a second experiment, we test if an *H. defensa* strain establishes a symbiosis more easily in aphid clones from a biotype that normally carries this symbiont species in nature compared to clones from a biotype that rarely carries *H. defensa*.

## Methods

### Study organisms.

Pea aphid clones were collected from one of four food plants (*Medicago sativa, Ononis spinosa, Lotus pedunculatus,* and *Lotus corniculatus*) between 2003 and 2012 in England, UK. It was previously confirmed that these aphid clones belonged to the particular plant-adapted populations from which they were collected and that they are phylogenetically clustered within the aforementioned pea aphid biotypes [9]. We will henceforth refer to these plant species as the aphid clones’ “native” food plants. Clonal aphid lines were established from one adult and reared in stock culture on broad bean (*Vicia faba*). *Vicia faba* is a plant species on which almost all pea aphid clones have been found to perform well, and it is often used for culturing pea aphids in the laboratory [26]. Aphid clones were screened for the seven facultative symbiont species known to commonly infect pea aphids using diagnostic PCR [9] on the 16S ribosomal RNA gene using species-specific primers (Additional File 1). Diagnostic PCRs were carried out by amplifying the symbiont 16S genes from aphid genomic DNA extractions using a “Touchdown” PCR (94 °C for 2 min; 11 cycles of 94 °C for 20s, 56 °C (dropping by 1 °C each cycle) for 50s, and 72 °C for 30s; 25 cycles of 94 °C for 2 min, 45 °C for 50s, and 72 °C for 2 min; a final 5 min extension period of 72 °C). All aphid clones used in this study (except those belonging to the *L. corniculatus* biotype) were confirmed to host only a single symbiont species, *Hamiltonella defensa*. Each of the three aphid clones harbored a unique *H. defensa* strain and each strain was confirmed to be within the clade that most commonly infects pea aphids associated with these three species of plants in nature [9, 27].

Facultative symbiont-free lines were created by the Godfray lab group at the University of Oxford using oral administration of selective antibiotics that cure only *H. defensa* and other facultative symbionts in the Enterobacteriaceae without affecting the obligate symbiont *Buchnera aphidicola* [28]. Cured lines were maintained at the University of Oxford for 50+ generations and then transferred to Vrije Universiteit Amsterdam. At Vrije Universiteit, stock cultures of infected and cured aphid clonal lines were maintained in a 15 °C room on a *V. faba* leaf within a petri dish, with the petiole in 1.5% agar solution. Clones were changed weekly to new leaves.

### Fitness effects during symbiont establishment in native and non-native aphid lineages.

Our first experiment sought to determine whether symbiont strains impose fitness costs when transferred to non-native hosts. For this we used two phylogenetically distinct *H. defensa* strains: strain 74 is the most common genotype associated with pea aphids feeding on *L. pedunculatus* plants and strain H218 is most commonly found in pea aphids feeding on *M. sativa* plants [9]. The two symbiont strains were infected into their native host and two non-native hosts using a full factorial design. Artificial infections were created by injecting symbiont-harboring hemolymph from a donor clone into a cured symbiont-free recipient clone. Single aphid clones from the host plants *L. pedunculatus* (clone 74), *M. sativa* (clone H218), and *O. spinosa* (clone 101), were infected with one of two different *H. defensa* strains (strains 74 and H218). Microinjection of hemolymph from donor to recipient produced six transfection treatments. Two treatments represented native associations (i.e. the symbiont strain 74 re-infected into the original “cured” aphid host 74, and the symbiont strain H218 re-infected into the original “cured” H218 aphid host), and four were non-native associations. To control for mortality due to hemolymph injections not caused by the presence of *H. defensa*, we injected all three aphid clones with hemolymph from a single aphid clone, the H218 clone, which had been cured of *H. defensa* (Additional File 2).

During microinjection, we removed a single leg from a known donor clone creating a bubble of hemolymph from the wound that was collected with a glass microinjection needle. Borosilicate glass capillary tubes (1.2 mm O.D., 0.94 mm I.D., 10 cm length) were heated and pulled using a Sutter P-87 micropipette puller to create a very fine tipped needle. In general, a single bubble of hemolymph was collected and injected into a recipient aphid. Under a dissecting scope, a known 2-day old recipient aphid was held in place with a paint brush and then injected into the “armpit” of the aphid’s back right leg. A successful injection involved little loss of hemolymph from the recipient aphid. Microinjections were completed in blocks, with 25 injection days over two months.

Post-injected aphids were cultured on *V. faba* leaves in 15-degree climate rooms. For each treatment we recorded: i) the proportion of injected individuals that survived to 7 days post-injection (9 days of age), whereby the aphid has molted to adulthood but has not yet reproduced, ii) the proportion of those that survived beyond 9 days that went on to reproduce, and iii) the proportion of individuals who passed the infection on to their offspring, as determined using diagnostic PCR of the 16S rRNA gene, on a single offspring selected several days after the adult started reproducing, following the methodology in [9]. Offspring of the injected aphids were screened for infection in their adult stage after they themselves became reproductive (see Table S2). Surviving infected combinations were maintained for fitness experiments for at least four generations, and transfected clones used in the fitness experiments originated from one original injected aphid per treatment. Each combination was sampled again prior to experiments to confirm that infections were not lost.

### Fitness effects of symbionts in different aphid clones.

Four of the six possible host-symbiont genotype combinations established and formed stable infections. They were used in the experiments for parasitoid protection and fecundity. One combination (symbiont H218 into host 101) did not establish due to the injected adults producing very few offspring that then themselves failed to reproduce (see Additional File 3). Another combination (symbiont 74 into host H218) was lost in culture.

#### Parasitoid protection.

We quantified the degree of protection conferred by each symbiont strain to each host aphid clone in a series of parasitoid trials. We exposed uninfected controls and transfected aphids to the parasitoid wasp *Aphidius ervi*, which uses aphids as a host to complete its reproductive cycle. Parasitoids oviposit in an aphid, and then the wasp larva develops and pupates within the aphid, forming a “mummy” from which an adult wasp emerges. If the wasp larva fails to develop, the aphid survives. A third outcome is that the aphid, and potentially wasp, dies, which we refer to as “non-mummified” aphid mortality.

Parasitoid mummies were obtained from Koppert Biocontrol (Berkel en Rodenrijs, The Netherlands) and transferred to a cage with honey and water. Parasitoid wasp trials took place three days after emergence to allow sufficient time for wasp mating following a modified protocol of [29] with blind treatment trials. One female wasp was transferred to a dish with 15 s-instar aphids. Aphids were moved individually to a new dish after observation of each oviposition. Each trial ran until the wasp was inactive for five minutes or all aphids had been stung. A new wasp was used for each replicate trial (*N* = 6), and the total number of aphids stung by each wasp was recorded. Only aphids that received an oviposition were maintained and cultured. Stung aphids were placed in dishes on a *V. faba* leaf in 1.5% agar and cultured in a 20-degree climate room. Aphids were transferred to a fresh leaf every three days. The number of mummies, surviving aphids, and dead aphids were counted 11 days after parasitism.

#### **Fecundity.**

To quantify the effect of symbiont infection on direct host fitness, we measured lifetime fecundity for each artificially infected host-symbiont treatment (*N* = 15 on each plant species). Lifetime fecundity was scored independently on both *V. faba* plants and the aphid’s native host plant. For the trials on native plants, aphid clones were transferred to their respective plant (*M. sativa, L. pedunculatus,* or *O. spinosa*) three generations prior to the fecundity assay. Fourth-instar aphids from each artificially infected treatment and symbiont-free treatment were transferred to a leaf in agar (one aphid per dish) and cultured in a temperature-controlled (15 °C) climate room. Each week, offspring from each adult was counted and discarded, and then the reproducing aphid was transferred to a fresh leaf. The number of offspring produced over the aphid’s lifetime was recorded.

### Symbiont establishment in aphid clones from two different biotypes.

We also tested whether a strain of *H. defensa* was more likely to establish in aphid clones from its original biotype (*L. pedunculatus*) compared to aphid clones from a biotype that does not normally carry *H. defensa* (*L. corniculatus*). *L. corniculatus*-adapted aphids have been shown to carry several secondary symbiont species in nature but most commonly harbor *Serratia symbiotica* [9]. Aphids from the *L. pedunculatus* and *L. corniculatus* biotypes are more closely related to each other than aphids belonging to the more distantly related *M. sativa* biotype [30].

Hemolymph from a single *H. defensa*-infected aphid clone 74 from the *L. pedunculatus* biotype was injected into each of six symbiont-free recipient clones: three clones from the *L. pedunculatus* biotype and three clones from the *L. corniculatus* biotype (*N* = 29–40 replicates per clone; see Additional File 4), using the same protocol as above. As in the previous experiment, the number of surviving, reproducing and infected aphids was recorded for each aphid clone. Injected aphids were cultured in an individual dish on a *V. faba* leaf. An adult aphid from the F1 generation of each injected line was sampled for infection using diagnostic PCR on the 16S rRNA gene of *H. defensa*. Control injections containing symbiont-free hemolymph were also administered to a single aphid clone of both biotypes.

### Data Analysis.

We examined i) the proportion of individual aphids that survived 7 days post injection and ii) the proportion of surviving aphids that went on to reproduce after injection with different strains of *H. defensa* and control injections containing only aphid hemolymph using Generalized Linear Models (GLM) with a binomial error distribution and logit link function. Explanatory variables for the survival and reproduction GLMs included i) symbiont treatment (no symbiont, symbiont strain H218, and symbiont strain 74), ii) aphid clone (H218, 74 and 101), and iii) an interaction between the two.

To determine if host-symbiont genotype interactions impact host fitness, we ran a second model that excluded the control injection containing no symbiont. In this analysis we tested for differences in survival and reproduction between native versus non-native genotype combinations using a GLM with three explanatory variables: i) symbiont strain (H218 and 74), ii) native versus non-native aphid clone, and iii) an interaction between the two. Here a “native combination” is the reinfection of a symbiont in the original “cured” host clone from which it was isolated.

We also used a GLM to determine if the symbiosis became established in the host and was successfully maternally transmitted by testing if the aphids still carried the symbionts in the generation following infection. Explanatory variables included symbiont strain, host clone, and the interaction between the two. For each injected aphid that survived to the F1 generation, a single offspring was sampled at adulthood to determine if it still carried the symbiont.

Parasitoid protection was quantified as the number of surviving aphids out of the total aphids stung using a GLM. We ran separate analyses for each symbiont strain in different aphid host backgrounds (symbiont 74 and symbiont H218 into their native and non-native aphid host genotype), including the control aphid clones uninfected with the symbiont. Explanatory variables included i) aphid clone, ii) presence/absence of symbiont and iii) an interaction between the two. An additional model examined the effect of symbionts 74 and H218 in the same aphid host background (host genotype 74). A GLM on the entire data set was also used to examine non-mummified aphid mortality between different host-symbiont combinations during parasitoid trials, and a second model verified that there was no observer bias during the parasitoid assays.

Differences in lifetime fecundity on the non-native universal host plant *V. faba* were tested with ANOVA. As with the previous analysis, the impact of symbiont infection on host fecundity was analyzed separately for each symbiont strain in a native and non-native host background, and then a separate analysis was conducted with both symbiont strains in a single host background (host 74). We used Bonferroni corrected *p*-values to account for multiple comparisons. Each of the three independent ANOVA models were repeated with aphids feeding on their native host plants.

We used a nested GLM to compare differences in the number of aphid clones within biotypes (clones from *L. corniculatus* versus clones from *L. pedunculatus*) that: i) survived 7 days after being infected with symbiont strain 74 (native to *L. pedunculatus*), and ii) if they still carried the symbiont in the F1 generation. For the survival analysis, the GLM had a binomial error distribution and logit link function with aphid clone nested within injection treatment as explanatory variable, where injection treatments include i) symbiont 74 injected into clones of their native biotype (*L. pedunculatus*), ii) symbiont 74 injected into clones from a non-native biotype or iii) a control injection with no symbiont. The presence of the symbiont in the F1 generation was analyzed with aphid clone nested within biotype as explanatory variables. Statistical analyses were conducted in JMP 13.0 statistical software (SAS Institute, Cary, NC, USA).

## Results

### Fitness effects during symbiont establishment in native and non-native aphid clones.

**Fig. 1:**
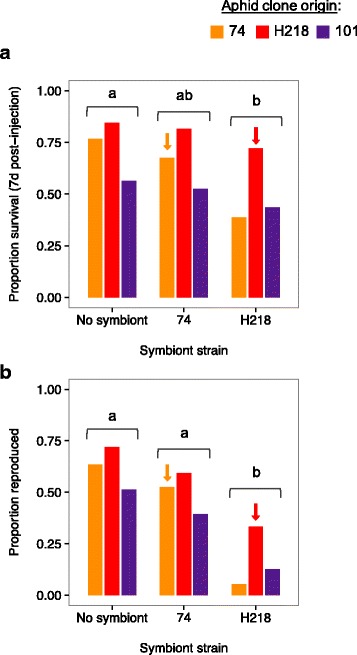
**a** Proportion survival of three aphid clones (*L. pedunculatus -* orange, *M. sativa -* red, and *O. spinosa -* purple) 7-days after injection of one of two *H. defensa* strains and a control injection containing only hemolymph with standard error (black bars). **b** Proportion aphids that survived 7-days and went on to reproduce after infection with the different symbiont strains or control treatment. Arrows indicate injections involving native host-symbiont combinations

Aphid survival and reproduction was influenced symbiont treatment (GLM symbiont treatment: host survival: χ^2^_2_ = 13.11, *p* = 0.001; host reproduction χ^2^_2_ = 66.61, *p* < 0.001); specifically, aphids infected with symbiont strain H218 had lower survival at 7 days after injection and had reduced reproduction when compared to the control injection (Fig. 1). In contrast, symbiont strain 74 had no impact on aphid survival or reproduction compared to the control. There was also an effect of aphid clone on survival, with clone H218 having greater survival overall (Aphid clone effect: χ^2^_2_ = 19.84, *p* < 0.001). There was no interaction between the three injection treatments and aphid clone (Interaction: χ^2^_4_ = 4.66, *p* = 0.32).

To determine if specific host and symbiont genotypes (GxG) interact in a way that differentially affects host fitness, we reanalyzed the data without the control injection, which lacked a symbiont. When comparing the native versus non-native host symbiont combinations, we find that aphids are more likely to survive (χ^2^_1_ = 8.40, *p* = 0.004) and reproduce (χ^2^_1_ = 8.32, p = 0.004) when harboring their native symbiont strain (Fig. 1, arrows). In addition, we find a significant host-symbiont GxG interaction for both survival (χ^2^_1_ = 4.45; *p* = 0.03) and reproduction (χ^2^_1_ = 7.17, *p* = 0.007), in that symbiont strain H218 caused higher mortality and reproductive failure in non-native aphid clones compared to the symbiont strain 74, which was essentially benign.

When the H218 symbiont strain was introduced into a non-native aphid clone, high rates of mortality occurred and few individuals managed to reach maturity. In rare cases, a few offspring were produced in surviving aphids, but these individuals typically suffered severely reduced reproduction to the F2 generation. However, in a single case we were able to produce a stable infection between the H218 strain and the non-native aphid host 74 (after *N* = 132 injections). We were unable to establish the H218 strain in the non-native aphid clone 101.

Injected aphids that survived to adulthood in the F1 generation showed no difference in the proportion of symbiont infection (Additional File 3), as establishment and maternal transmission of the symbiont was not influenced by symbiont strain (GLM: χ^2^_1_ = 0.29, *p* = 0.59), host clone (χ^2^_2_ = 1.21, *p* = 0.55), or an interaction between the two (χ^2^_2_ = 2.38, *p* = 0.32). A full account of the number of aphids injected that survived to produce stable infections is presented in Additional File 3.

### Fitness assays.

#### **Parasitoid protection.**

**Fig. 2:**
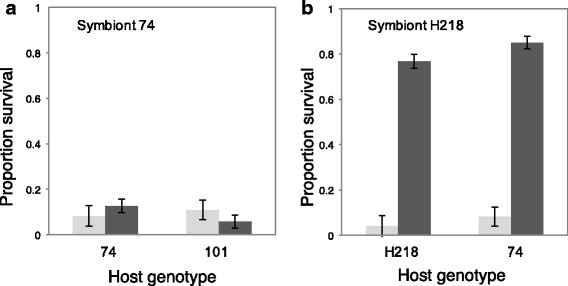
Parasitoid resistance conferred by *H. defensa* strains in different host backgrounds, measured as the proportion of surviving aphids out of total number stung. **a** Mean survival in symbiont-free hosts (light grey columns) from two genotypes compared to survival in hosts infected with the *L. pedunculatus* strain of *H. defensa* (dark grey columns). **b** Mean survival in symbiont-free hosts (light grey columns) from two genotypes compared to survival in hosts infected with the *M. sativa* strain of *H. defensa* (dark grey columns). Columns on the left of each panel represent comparisons of uninfected hosts to native host-symbiont genotype combinations and columns on the right are comparisons to non-native combinations. Error bars denote standard error

Symbiont strain 74 did not provide protection against the parasitoid *A. ervi* when harbored by either its 74 host aphid or a non-native host (χ^2^_1_ = 2.20, *p* = 0.53) (Fig. 2a). However, symbiont strain H218 conferred a high degree of protection to both its native host clone (H218) and non-native host clones (74) (χ^2^_1_ = 181.80, *p* < 0.001) (Fig. 2b). Comparison of both symbionts within aphid clone 74 confirms that the H218 strain of *H. defensa* provided significantly higher protection from parasitoids (χ^2^_2_ = 111.42, p < 0.001) than strain 74.

There was no difference in non-mummified aphid mortality following parasitoid attack when aphid clone 74 carried either of the two *H. defensa* strains or was uninfected (χ^2^_2_ = 3.51, *p* = 0.17). Likewise, there was no difference in mortality when different aphid clones carried the symbiont strain 74 or were uninfected (χ^2^_3_ = 4.96, *p* = 0.18). There was higher mortality after wasp oviposition in aphid clones infected with the symbiont strain H218 compared to uninfected controls (χ^2^_1_ = 4.26, *p* = 0.04); however, there was no interaction between aphid clones and whether they were infected by the symbiont or not (χ^2^_1_ = 0.19, *p* = 0.66). Therefore, mortality was not significantly different among the host-symbiont genotype combinations. To be conservative, mortality was included into the metric for parasitoid protection illustrated in Fig. 2 (surviving aphids over total number stung). When non-mummified dead aphids are excluded, the symbiont strain H218 provided hosts with 100% protection from parasitoids in both native and non-native aphid clones. There was no effect of observer on the oviposition trials (GLM: χ^2^_1_ = 2.29, *p* = 0.12).

#### **Fecundity.**

**Fig. 3:**
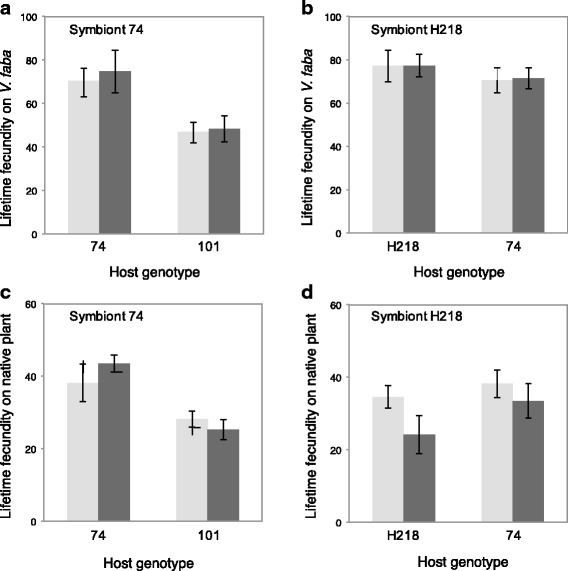
Mean lifetime fecundity of aphids feeding on broad bean (*V. faba*) (**a**, **b**) and on native host plants (**c**, **d**). Aphids infected with symbionts (dark grey columns) are compared to the same symbiont-free aphid clone (light grey columns). Mean fecundity of aphid clones carrying the *L. pedunculatus* symbiont strain are presented feeding on broad bean (*V. faba*) (**a**) and their native host plant (**c**). Mean fecundity of aphid clones harboring the *M. sativa* symbiont strain are also presented for aphids feeding on broad bean (*V. faba*) (**b**) and their native host plant (**d**). Native symbiont-host genotype comparisons are on the left of each panel. Error bars denote standard error

We found that neither symbiont strain had an impact on aphid fecundity when feeding on *V. faba* leaves, regardless of whether they were harbored by a native or non-native aphid clone (symbiont strain 74 effect: F_1,59_ = 0.17, *p* = 1.00; symbiont*host interaction F_1,59_ = 0.04, *p* = 1.00; symbiont strain H218 effect F_1,64_ = 0.009, p = 1.00; symbiont*host interaction F_1,64_ = 0.004, p = 1.00) (Fig. 3). Aphid clone 74 did, however, have higher intrinsic fecundity than clone 101 (host effect: 74–101 F_1,59_ = 13.50, *p* = 0.002; 74-H218 F_1,64_ = 1.01, *p* = 0.64) (Fig. 3 a, b). We also found no difference in fecundity when the same aphid clone harbored either of the symbiont strains or was uninfected (Symbiont effect: F_2,44_ = 0.10, p = 1.00).

When feeding on their native host plants, we found no effect of symbiont presence or strain on aphid fecundity (symbiont strain 74 effect, F_1,60_ = 0.14, p = 1.00; symbiont*host interaction F_1,60_ = 1.44, *p* = 0.46; symbiont strain H218 effect, F_1,60_ = 3.81, *p* = 0.12; symbiont*host interaction F_1,60_ = 0.54, *p* = 0.94), and the intrinsic difference in the fecundity of the 74 and 101 aphid clones remained (Host effect: 74–101 F_1,60_ = 16.81, p = 0.002;74-H218*,* F_1,60_ = 2.75, *p* = 0.20) (Fig. 3 c, d). Again, there was no difference in a single clone harboring different symbiont strains (F_2,45_ = 1.55, *p* = 0.44).

### Symbiont establishment in aphid clones from different plant-adapted biotypes.

**Fig. 4:**
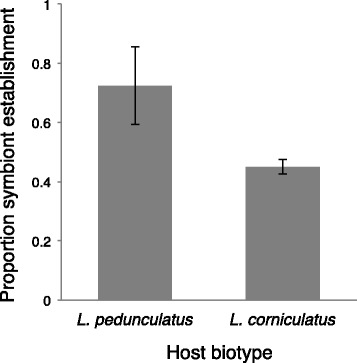
Mean establishment of *H. defensa* in three aphid clones belonging to the *L. pedunculatus* biotype and three from the *L. corniculatus* biotype with standard error (black bars). The *L. pedunculatus* biotype frequently harbors *H. defensa* in field populations, whereas the *L. corniculatus* biotype does not

There was no difference in the survival of aphid clones from *L. pedunculatus* and *L. corniculatus* biotypes when injected with the *L. pedunculatus* strain of *H. defensa* and a control injection with no symbiont (Aphid clone: χ^2^_5_ = 5.87, *p* = 0.31; Injection treatment (Biotype): χ^2^_2_ = 2.99, *p* = 0.22) (see Additional File 4). However, aphid clones belonging to *L. pedunculatus* biotype were significantly more likely to establish a symbiosis with *H. defensa* compared to clones from the *L. corniculatus* biotype (Biotype effect χ^2^_1_ = 9.7, *p* = 0.002). There was also a significant variation among clones within biotype (Biotype:clone χ^2^_4_ = 18.65, *p* < 0.001). Establishment success among clones within the *L. pedunculatus* biotype varied from 100% in one clone to 47.4% in another, which was in host 74 the symbiont’s native clone. On average, *H. defensa* established in 72.5% (±13.2%) of the injected individuals in aphid clones from *L. pedunculatus* compared to 45.0% (±2.5%) of the individuals from *L. corniculatus* clones when sampled in the F1 generation after infection (Fig. 4).

## Discussion

Our study provides evidence of incompatibilities between host and symbiont genotypes that can influence the horizontal transfer of symbiotic microbes among populations of insects. We show that a strain of the symbiont *H. defensa* can actually be pathogenic and inhibit reproduction when initially transferred into non-native host aphid clones, while another *H. defensa* strain is not virulent to new hosts. However, once symbionts pass this potential barrier and became established, they can transfer an important adaptive trait, like parasitoid protection, on to the recipient host. In addition, we show that *H. defensa* is more likely to establish within clones of a biotype that normally harbors this symbiont in nature compared to clones from a biotype that does not normally carry this microbe. These findings suggest that host-symbiont genotype compatibility is an understudied yet potentially significant force that may affect distributions of facultative symbionts in natural populations of hosts.

We found that both *H. defensa* strains were less likely to cause mortality in their native hosts compared to non-native strains; however, the two strains differed significantly in how virulent they were to non-native aphid clones. Compared to control injections that contained only hemolymph, the three aphid clones experienced minimal costs when infected with strain 74 of *H. defensa*, while strain H218 caused significantly higher mortality and inhibited offspring production in both non-native host clones. The detrimental effect on aphid survival and reproduction did not occur when hemolymph was injected from an H218 aphid that had been cured of *H. defensa*, demonstrating that this specific strain of *H. defensa* was likely responsible for the negative impact on host fitness. Although the H218 symbiont strain was more virulent, it was much less so when in its native host background, indicating a greater degree of co-adaptation to its native aphid lineage. Thus, harboring a non-adapted symbiont can result in negative fitness consequences for the host.

Evidence that symbiont establishment can have detrimental effects on non-native host species has been demonstrated in several other systems. Certain strains of the reproductive manipulating symbiont *Wolbachia* can reduce host fecundity when introduced to *Drosophila* species that do not typically harbor this microbe [19]. In isopods, infection of non-native *Wolbachia* strains also reduced fertility in recipient hosts [20] and caused nervous system disorders and mortality [21]. Similarly, certain strains of the normally mutualistic bacteria *Xenorhabdus nematophila* inhibited reproduction when transferred to non-native host nematodes [22]. The beneficial gut bacteria of two related *Nasonia* species show high host-specificity, so much so that they caused lethality in F2 hybrid wasps [31]. Hybrids of two mice subspecies also suffered fitness reductions due to genetic incompatibilities caused by harboring gut microbes that are adapted to either mouse subspecies [32]. These studies highlight the potential costs associated with acquiring symbiotic bacteria that hosts are not adapted to.

However, as we have shown, there can be significant variation in the consequences of housing new symbionts; some may be highly pathogenic while others may have no negative fitness consequences at all (e.g. [33]). We find that even *within* a single host species, symbiont isolates can vary dramatically in their degree of pathogenicity, and their potential to damage different aphid clones depends on whether the host has had experience carrying the symbiont isolate or not (e.g. nativeness). It will be interesting to see whether the fitness costs associated with *Hamiltonella* transfer are greater between pea aphid biotypes compared to within the same biotype. Indeed, determining whether the frequency of harmful *Hamiltonella* isolates, and the severity of the costs, differs within or between biotypes is key to understanding how these interactions influence the presence of symbionts in nature. Our findings highlight the importance of genotype interactions in the evolution of mutualisms and demonstrate that presumed “mutualistic” bacteria such as *H. defensa* are not universally beneficial and can even cause severe fitness costs when interacting with different host lineages within a single insect species.

After an initial establishment period of two host generations, the cost of carrying the *M. sativa* strain of *H. defensa* ameliorated, and there were no negative fitness consequences in subsequent generations. This indicates that pathogenicity is a transient effect, and if a symbiont strain has the potential to harm the host, it is most likely to occur immediately after being acquired through horizontal transfer. Amelioration of the cost associated with symbiont establishment in a new host has also been observed in the *popcorn* strain of *Wolbachia*, where host fecundity and lifespan declined following transfection but improved in each subsequent generation [19]. *Spiroplasma* infection into a novel host species of *Drosophila* also had strong fecundity costs that were ameliorated 17 generations after transfection [34]. Bacteria also undergo a period of compensatory adaptation when acquiring new symbiotic plasmids, which eventually stabilizes plasmids by reducing the cost of their carriage [35]. Taken together with our findings, this demonstrates that for symbiont strains with the potential to be virulent, fitness consequences are most likely to occur when a symbiont is first introduced to a new host, but if the host survives the initial infection, the symbiosis can stabilize and persist within the new lineage.

It is unclear what causes *H. defensa*’s virulence when introduced to new hosts, but there are several potential sources of pathogenicity. *H. defensa* has evolved from a pathogenic ancestor and its genome contains abundant homologs of toxin and virulence genes including type-3 secretion systems and regulatory genes involved in timing their expression [36]. These loci are thought to be important for establishing a symbiosis with naïve hosts, but they are also used to invade host cells and evade host immune responses and therefore have the potential to be harmful. In addition, most *H. defensa* strains harbor the APSE phage that encodes toxins that are pathogenic to eukaryotic cells and are involved in protecting hosts from attack by parasitoid wasps [37]. An alternate hypothesis is that the symbiont or phage may interact detrimentally with the primary symbiont *Buchnera*; for instance, the secondary symbiont *Serratia symbiotica* reduced growth and reproduction of host aphids through such an interaction [38]. Differentiating between these hypotheses will require manipulated studies that explore the molecular interactions between the symbionts, host, and phage in vivo.

Previous research demonstrated that *H. defensa* can provide fitness benefits in different host backgrounds by protecting hosts from attack by parasitoid wasps [24, 39]. We hypothesized that certain symbiont isolates may provide certain host clones with greater fitness benefits than others, which could explain their persistence in particular host lineages. However, our results reject this hypothesis. Carrying a native or non-native symbiont strain did not affect the degree of symbiont-conferred protection from parasitoids or host fecundity. Although there was no effect of *H. defensa* strain on aphid fecundity, there were significant differences in the protective benefits provided by the two symbiont strains. Strain H218 of *H. defensa* conferred 100% protection from parasitoids in both its native and non-native hosts, whereas strain 74 conferred weak protection compared to symbiont-free clones, although it has been shown to be protective against another species of parasitoid wasp [29, 40]. The highly protective strain H218 is also the one that caused higher host mortality and inhibited reproduction when initially transferred to non-native hosts, whereas the weakly protective strain 74 did not cause significant mortality in any of the aphid clones. Future studies will explore how variation in virulence and protection influences the spread of symbionts under different selective pressures. Strain-level differences in parasitoid protection of *H. defensa* have been recently quantified [29], but our study is the first to show this symbiont’s potential to cause host mortality and inhibit offspring production during the initial infection period.

It has been proposed that niche-specific benefits conferred by symbionts to their hosts may explain why symbionts are found at high frequencies in certain insect populations [10, 11]. Our results suggest that incompatibilities between host and symbiont genotypes that make up different host populations may be another force limiting the spread of symbionts among populations of insects and possibly other taxa where facultative symbionts are common. We found that related pea aphid clones belonging to the major clade associated with the plant *L. pedunculatus*, which commonly carry *H. defensa*, were more likely to establish a symbiosis with this symbiont compared to aphids associated with the plant *L. corniculatus*, which rarely harbor this symbiont in nature. This indicates that the limitations to horizontal transmission of symbionts exist not only between host lineages, but also between populations of hosts adapted to different plant species. In future field-based studies, it will be interesting to see how important establishment biases are in shaping the frequencies of symbionts we observe in nature populations of pea aphids.

A recent study by Parker et al. (2017) demonstrated that aphid clones from biotypes that normally harbor the symbiont *Regiella insecticola* are more likely to accept and maintain a symbiosis with this microbe following transfections [18]. These results in combination with our own suggest a more broadly important phenomenon that is likely to affect transfer of many different symbiont species between pea aphid biotypes. *H. defensa* strains are more likely to transfer between closely related aphid species, suggesting phylogenetic distance between host species can limit the horizontal transmission of facultative symbionts [16]. Similarly, horizontal transfer success of the reproductive parasites *Wolbachia* and *Spiroplasma* between host species has also been shown to be dependent on relatedness to its native host species [17, 41]. We find that even within a single insect species, certain populations of hosts are more likely to establish symbioses with facultative microbes, indicating some form of preadaptation to carrying symbionts that may impact the establishment and maintenance of facultative symbionts in different populations of hosts.

Many studies have shown that facultative symbionts can be non-randomly distributed across host populations in nature, and these patterns are particularly common in genetically differentiated insect races or biotypes that occupy different feeding niches. Different genetic groups of the whitefly species complex *Bemisia tabaci* have different S-symbiont statuses [7, 42]; for example, almost all individuals within the MED species subgroup Q1 harbor *H. defensa*, while MED Q2 and MED Q3 individuals do not [43, 44]. In Drosophilid species, *Wolbachia* infection is more common in the subgenus *Sophophora* than in the subgenus *Drosophila* [45]. In the chestnut weevil *Curculio sikkimensis*, infections by *Sodalis* and *Rickettsia* symbionts are higher in populations feeding on acorns than on chestnuts [8]. Five out of six aphid species investigated have uneven distributions of facultative symbionts in populations adapted to different species of plants, and this occurs globally in at least two aphid species [9, 26, 46]. Although it is tempting to consider the non-random patterns of facultative symbionts as a product of differing host ecologies, our results suggest that genetic mismatches during horizontal transmission may be an understudied mechanism explaining their distributions. Our results demonstrate that host and symbiont genotypes can interact in a way that can both cause pathogenicity to the host and prevent the successful establishment of a symbiosis; however, the current study does not quantify the frequency of these “conflictive” versus “non-conflictive” symbiont genotypes. Future studies are needed to assess the strength of genotype incompatibility as a force explaining symbiont distributions within and between populations and to determine if pathogenicity in *Hamiltonella* is a wide-spread or localized phenotype.

## Conclusions

Our study has shown that genetic incompatibilities can limit the spread of symbiotic bacteria, which may help explain the presence of these microbes in certain host populations. Although multiple competing factors including genetics and ecology likely contribute to the transmission of facultative symbionts, we have shown that preadaptation of a symbiont to its host is necessary in order to form a new symbiotic relationship and that incompatibility can even result in host mortality. Only once the barrier to establishment is surpassed can the advantageous traits carried by symbionts be bestowed on the host. Therefore, the model of freely shuttled adaptations as a driving force of symbiont distribution in nature is likely oversimplified. Factors that hinder and promote spread of facultative symbionts will exist concurrently in any system; however, we have shown that genotype incompatibilities can prevent the horizontal transfer of symbionts and therefore has the potential to limit the unconstrained spread of these potentially beneficial microbes.

## Additional files

Additional file 1: Diagnostic symbiont species-specific primer sequences. (PDF 70 kb)

Additional file 2: Production of artificial infection treatments by microinjection of *H. defensa*-containing hemolymph from two donor aphid clones into three symbiont-free recipient aphid clones, named according to their host plant (*L. pedunculatus*, *M. sativa*, or *O. spinosa*), producing two native symbiont strain by host aphid clone associations (“native”) and four non-native associations (“non-native”), plus three control treatments of recipient clones injected with hemolymph from a single *M. sativa* clone that had been cured of *H. defensa* (“control”). (PDF 54 kb)

Additional file 3: Survival, reproduction, and establishment of *H. defensa* strains in different host aphid clones. Aphids that have been cured of *H. defensa* have a “c” prior to the clone number. (PDF 85 kb)

Additional file 4: Survival, reproduction and establishment of a *Lotus pedunculatus* strain of *H. defensa* in aphid clones from two different biotypes. All injected recipient clones were either naturally free of secondary symbionts or were selectively cured of their secondary symbionts (“c” prior to the clone number). (PDF 87 kb)

## Abbreviations

*A. ervi*: *Aphidius ervi* (parasitoid wasp)
GLM: Generalized linear model
*H. defensa*: *Hamiltonella defensa* (facultative symbiont)
*L. corniculatus*: *Lotus corniculatus* (trefoil plant)
*L. pedunculatus*: *Lotus pedunculatus* (trefoil plant)
*M. sativa*: *Medicago sativa* (alfalfa plant)
*O. spinosa*: *Ononis spinosa* (restharrow plant)
*V. faba*: *Vicia faba* (broad bean plant)
