# Supplementary material for: Symbiont interactions with non-native hosts limit the formation of new symbioses
Source: BMC Evol Biol. 2018 Mar 12;18:27. doi: 10.1186/s12862-018-1143-z (PMC5848548; doi:10.1186/s12862-018-1143-z)
Supplement: Supplementary file 1 — Diagnostic symbiont species-specific primer sequences. (PDF 70 kb) [file 12862_2018_1143_MOESM1_ESM.pdf]

**Symbiont species**	**Gene**	**Primer names**	**Forward primer (5' to 3')**	**Reverse primer (5' to 3')**
Hamiltonella defensa	16S rRNA	10F - T419R	AGTTTGATCATGGCTCAGATTG	AAATGGTATTSGCATTTATCG
Regiella insecticola	16S rRNA	10F - R443R	AGTTTGATCATGGCTCAGATTG	GGTAACGTCAATCGATAAGCA
Serratia symbiotica	16S rRNA	rRNA 16SA1 -16S.S2R	AGAGTTTGATCMTGGCTCAG	TTTGAGTTCCCGACTTTATCG
X-type	16S rRNA	10F - X420R	AGTTTGATCATGGCTCAGATTG	GCAACACTCTTTGCATTGCT
Rickettsia sp.	16S rRNA	rRNA 16SA1 -16S.Ri2R	AGAGTTTGATCMTGGCTCAG	TTTGAAAGCAATTCCGAGGT
Spiroplasma sp.	16S rRNA	rRNA 16SA1 -16S.SpR	AGAGTTTGATCMTGGCTCAG	ATCATCAACCCTGCCTTTGG
Rickettsiella sp.	16S rRNA	RCL16S-211F - RCL16S-470R	GGGCCTTGCGCTCTAGGT	TGGGTACCGTCACAGTAATCGA

Additional File 1. Diagnostic symbiont species-specific primer sequences
