# Supplementary material for: Symbiont interactions with non-native hosts limit the formation of new symbioses
Source: BMC Evol Biol. 2018 Mar 12;18:27. doi: 10.1186/s12862-018-1143-z (PMC5848548; doi:10.1186/s12862-018-1143-z)
Supplement: Supplementary file 2 — Production of artificial infection treatments by microinjection of H. defensa-containing hemolymph from two donor aphid clones into three symbiont-free recipient aphid clones, named according to their host plant (L. pedunculatus, M. sativa, or O. spinosa), producing two native symbiont strain by host aphid clone associations (“native”) and four non-native associations (“non-native”), plus three control treatments of recipient clones injected with hemolymph from a single M. sativa clone that had been cured of H. defensa (“control”). (PDF 54 kb) [file 12862_2018_1143_MOESM2_ESM.pdf]

	Donor (symbiont)		
Recipient (host)	*Lotus pedunculatus*	*Medicago sativa*	No symbiont
Lotus pedunculatus	**native**	**non-native**	**control**
Medicago sativa	**non-native**	**native**	**control**
Ononis spinosa	**non-native**	**non-native**	**control**

Additional File 2. Production of artificial infection treatments by microinjection of *H. defensa*-containing hemolymph from two donor aphid clones into three symbiont-free recipient aphid clones, named according to their host plant (*L. pedunculatus*, *M. sativa*, or *O. spinosa*), producing two native symbiont strain by host aphid clone associations ("native") and four non-native associations ("non-native"), plus three control treatments of recipient clones injected with hemolymph from a single *M. sativa* clone that had been cured of *H. defensa* ("control").
