# Supplementary material for: Symbiont interactions with non-native hosts limit the formation of new symbioses
Source: BMC Evol Biol. 2018 Mar 12;18:27. doi: 10.1186/s12862-018-1143-z (PMC5848548; doi:10.1186/s12862-018-1143-z)
Supplement: Supplementary file 3 — Survival, reproduction, and establishment of H. defensa strains in different host aphid clones. Aphids that have been cured of H. defensa have a “c” prior to the clone number. (PDF 85 kb) [file 12862_2018_1143_MOESM3_ESM.pdf]

Donor aphid clone (symbiont strain)	Recipient aphid clone (biotype)	Native/ Non-native	# aphids injected	# survival to ~7 days	% survival to ~7 days	# survival to reproduction (F1 generation)	% survival that reproduced	# survival to reproduction (F2 generation)	# sampled for infection	# positive infection	% infected
H218 (*M. sativa*)	c74 (*L. pedunculatus*)	Non-native	132	51	38.6	7	13.7	2	2	1	50.0
H218 (*M. sativa*)	cH218 (*M. sativa*)	Native	36	26	72.2	12	46.1	11	11	5	45.5
H218 (*M. sativa*)	c101 (*O. spinosa*)	Non-native	55	24	43.6	7	29.2	1	1	0	0.00
74 (*L. pedunculatus*)	c74 (*L. pedunculatus*)	Native	40	27	67.5	21	77.8	20	19	9	47.4
74 (*L. pedunculatus*)	cH218 (*M. sativa*)	Non-native	27	22	81.5	16	72.7	14	14	6	42.9
74 (*L. pedunculatus*)	c101 (*O. spinosa*)	Non-native	38	20	52.6	15	75.0	8	8	6	75.0
cH218 (cured)	c74 (*L. pedunculatus*)	N/A	30	23	76.7	19	82.6	19			
cH218 (cured)	cH218 (*M. sativa*)	N/A	32	27	84.4	23	85.2	23			
cH218 (cured)	c101 (*O. spinosa*)	N/A	39	22	56.4	20	90.9	20			

Additional File 3. Survival, reproduction, and establishment of *H. defensa* strains in different host aphid clones. Aphids that have been cured of *H. defensa* have a "c" prior to the clone number.
