# Supplementary material for: Symbiont interactions with non-native hosts limit the formation of new symbioses
Source: BMC Evol Biol. 2018 Mar 12;18:27. doi: 10.1186/s12862-018-1143-z (PMC5848548; doi:10.1186/s12862-018-1143-z)
Supplement: Supplementary file 4 — Survival, reproduction and establishment of a Lotus pedunculatus strain of H. defensa in aphid clones from two different biotypes. All injected recipient clones were either naturally free of secondary symbionts or were selectively cured of their secondary symbionts (“c” prior to the clone number). (PDF 87 kb) [file 12862_2018_1143_MOESM4_ESM.pdf]

| Donor aphid clone (symbiont strain) | Recipient aphid clone (biotype) | # aphids injected | # survival to ~7 days | % survival to ~7 days | # survival to reproduction (F1 generation) | % survival that reproduced | # survival to reproduction (F2 generation) | # sampled for infection | # positive infection | % infected |
|---|---|---|---|---|---|---|---|---|---|---|
| 74 (*L. pedunculatus*) | c74 (*L. pedunculatus*) | 35 | 21 | 60.0 | 19 | 90.5 | 19 | 19 | 9 | 47.4 |
| 74 (*L. pedunculatus*) | c132 (*L. pedunculatus*) | 30 | 22 | 73.3 | 22 | 100 | 22 | 20 | 14 | 70.0 |
| 74 (*L. pedunculatus*) | 672 (*L. pedunculatus*) | 35 | 24 | 68.6 | 21 | 87.5 | 21 | 20 | 20 | 100 |
| 74 (*L. pedunculatus*) | 663 (*L. corniculatus*) | 33 | 26 | 78.8 | 23 | 88.5 | 23 | 20 | 9 | 45.0 |
| 74 (*L. pedunculatus*) | 664 (*L. corniculatus*) | 35 | 29 | 82.9 | 25 | 86.2 | 25 | 20 | 8 | 40.0 |
| 74 (*L. pedunculatus*) | 451 (*L. corniculatus*) | 40 | 26 | 65.0 | 23 | 88.5 | 23 | 20 | 10 | 50.0 |
| c74 (cured) | c74 (*L. pedunculatus*) | 29 | 23 | 79.3 | 22 | 95.7 | | | | |
| 663 (symbiont-free) | 663 (*L. corniculatus*) | 33 | 26 | 78.8 | 25 | 96.2 | | | | |

Additional File 4. Survival, reproduction and establishment of a *Lotus pedunculatus* strain of *H. defensa* in aphid clones from two different biotypes. All injected recipient clones were either naturally free of secondary symbionts or were selectively cured of their secondary symbionts ("c" prior to the clone number).
